# Protective Effects of Hydroalcoholic Extract of *Rosa canina* Fruit on Vancomycin-Induced Nephrotoxicity in Rats

**DOI:** 10.1155/2021/5525714

**Published:** 2021-05-28

**Authors:** Hossein Sadeghi, Ehsan Karimizadeh, Heibatollah Sadeghi, Esmaeel Panahi kokhdan, Mahboubeh Mansourian, Kazem Abbaszadeh-Goudarzi, Mansoureh Shokripour, Arash Asfaram, Amir Hossein Doustimotlagh

**Affiliations:** ^1^Medicinal Plants Research Center, Yasuj University of Medical Sciences, Yasuj, Iran; ^2^Student Research Committee, Yasuj University of Medical Sciences, Yasuj, Iran; ^3^Cellular and Molecular Research Center, Sabzevar University of Medical Sciences, Sabzevar, Iran; ^4^Department of Pathology, School of Medicine, Shiraz University of Medical Sciences, Shiraz, Iran

## Abstract

Vancomycin-induced nephrotoxicity (VIN) has been reported to occur in 5–35% of recipient patients. The aims of the study were to evaluate protective effects of *Rosa canina* (RC) on VIN in rats. Rats were randomly divided into five groups as follows: control group I, group II (received VAN 400 mg/kg/day, every 12 h at doses of 200 mg/kg/day, for 7 consecutive days), group III (VAN + RC 250 mg/kg/day, for 7 consecutive days), group IV (VAN + RC 500 mg/kg/day, for consecutive days), and group V (received RC 500 mg/kg/day, for consecutive 7 days). On the eighth day after anesthetizing the animals, blood samples were taken from the heart, and then, the kidneys were removed to investigate kidney function, oxidative stress, and histopathological marker. Also, the chemical composition of RC extract was identified by GC-MS analysis. Oral dose of 500 mg/kg RC extract significantly reduced the serum levels of blood urea nitrogen (BUN), creatinine (Cr), malondialdehyde (MDA), and nitric oxide (NO) and also the kidney tissue MDA, protein carbonyl, and NO metabolites (nitrite) levels compared to the VAN-treated group (*P* < 0.05). Based on histopathological analysis, RC extract at the dose of 500 mg/kg inhibited the destructive effects of VAN on kidney tissues. GC-MS analysis indicated that the main compositions were found to be lactose (21.96%), 3-t-butyloxaziridine (20.91%), and 5-oxymethylfurfurole (16.75%). The results indicated that oral administration of RC was able to reduce VAN-induced nephrotoxicity in rats, possibly through antioxidant pathways.

## 1. Introduction

Vancomycin is a widely used antibiotic that acts as an inhibitor in the synthesis of bacterial cell wall to treat infections caused by methicillin-resistant *Staphylococcus aureus* (MRSA) and methicillin-resistant *Staphylococcus epidermidis* (MRSE) [1].

VAN is mostly excreted through the kidneys, and that is why it has potentially lethal side effects leading to nephrotoxicity. These side effects limit its administration and efficacy [2]. Nephrotoxicity duo to VAN therapy was reported in 5–30% of patients [3], while this can increase to 20–35% when combined with an aminoglycoside antibiotic [2, 4]. On average, nephrotoxicity developed between 4 and 17 days after VAN therapy [5]. Although the exact mechanism of VAN-induced nephrotoxicity is not well understood, studies have suggested that oxidative stress [4, 6], inflammatory process, and apoptosis may be involved in the pathogenesis of renal toxicity of VAN [2]. As mentioned in the literature, oxidative damage is the consequence of unevenness amongst reactive oxygen species (ROSs) and antioxidants leading to cellular harm [7]. The production of ROS leads to oxidative stress associated with mitochondrial dysfunction and antioxidant system disturbance [8]. In renal cells, by increasing ATP concentration and stimulating oxygen consumption, it is reported that VAN can raise ROS production [9]. However, ROS production is considered to be a vital reason for damage to renal cell membranes resulting in lipid peroxidation, protein denaturation, and the reduction of antioxidative enzymes, including superoxide dismutase and catalase [3, 10, 11]. Additionally, some antioxidants including zingerone [12], curcumin [13], thymoquinone [3], caffeic acid phenethyl ester (CAPE), and N-acetylcysteine [14] have shown protective effects against VAN-induced renal toxicity.

*Rose canina* (RC), an erect shrub, is a medicinal herb that is widely distributed in Europe, western Asia, and northeastern Africa, backdated to Hippocrates's time. During World War II, the syrup of RC fruits was common to avoid scurvy because of a large amount of ascorbic acid [15, 16]. In some countries, RC is used as a traditional medicine to treat a wide range of diseases, including vitamin C deficiency, diabetes, poor peripheral circulation, gout, hemorrhoids, influenza, common cold, biliary disorders, gallstones, rheumatoid arthritis, osteoarthritis, rheumatism, and dysfunction in the gastrointestinal and urinary system [17, 18]. However, it is known that RC has numerous active components such as phenolic acids, carotenoids, tocopherols, proanthocyanidins, pectin, tannins, flavonoids, unsaturated and polyunsaturated fatty acids, phospholipids, minerals, galactolipids, and vitamins (mainly C, B1, B2, B3, K, and E) [17, 19, 20]. Based on previous findings, quercitrin, hyperoside, vitamin C quercetin-3-o-glucoside, beta-sitosterol, folic acid, and beta-carotene are the main components in the rose hip extract [18, 21]. Some pharmacological activities of RC such as anti-inflammatory [22], antioxidant [23, 24], antiproliferative [25], and antiobesity [26] have been confirmed. More importantly, it has been reported that protective effects of RC in renal failure are induced by ischemia/reperfusion injury [16]. According to our knowledge, there is no appropriate and definitive treatment to reduce the renal toxicity of VAN. Also, it has been reported that oral consumption of *Rosa canina* hip extract reduced the kidneys toxicity in the model of ischemic/reperfusion in rats [16]. Therefore, the current study was designed to investigate the protective effect of oral treatment with hydroalcoholic extract of RC fruits against VAN-induced nephrotoxicity in rats.

## 2. Materials and Methods

### 2.1. Plant Material

Ripe RC fruit was collected in June 2017, from the suburbs of Yasuj, Iran, which was evaluated by Dr. A. Jafari (Botany Department, Natural Resource and Animal Husbandry Research Centre, Yasuj University, Yasuj, Iran) [27]. The seeds were cleaned, and dust and soil were removed by using distilled water. Then, the pericarp of rose hip was dried in the shade and then pulverized using scissors.

#### 2.1.1. Preparation of Extract

Plant parts, powdered RC, were macerated in EtOH-H_2_O (50/50, v/v) at room temperature for 48 h, and then, 50% ethanol was added to remaining material for 24 h and consequently percolated through a column. Finally, the extract solution was concentrated using a rotary evaporator (Hyedolph, type: HeizbadHei-VAP, Germany) under reduced pressure at 40°C. It should be noted that the extract was stored at −20°C [28, 29].

### 2.2. Chemicals and Reagent

Some materials were purchased from Sigma Chemical Co. (St. Louis, MO, USA), including VAN, thiobarbituric acid (TBA), acetonitrile, 5,5′-dithiols-2-nitrobenzoic acid (DTNB), and ethylenediaminetetraacetic acid (EDTA). All other materials were obtained from Merck (Germany) such as trichloroacetic acid (TCA), 2, 4-dinitrophenylhydrazine (DNPH), and formaldehyde. Of note, all chemicals and reagents were used in the experiment and had analytical grade.

### 2.3. Animals and Experimental Conditions

Experimental procedures were accepted by the Ethics Committee of Yasuj University of Medical Sciences (IR.Yums.REC.1395.115). Based on the “Principles of Laboratory Animal Care” (NIH Publication No. 86-23), the animals were managed by using guidelines provided for the care of laboratory animals. About thirty-five male Wistar rats (weight 205 ± 25 g) were provided from the Razi Institute of Iran (Tehran, Iran). Under constant temperature and humidity (24 ± 2°C, 55 ± 60%) on a controlled light-dark cycle with free access to food and water, animals were kept. The animals were adapted before the experiment for one week. After the adaptation, rats were randomly divided into five groups of seven each as follows:

  Group I (control group): injected normal saline intraperitoneally (I.P) for 7 for consecutive days
  Group II (VAN group): VAN was injected I.P every 12 h at doses of 200 mg/kg/day for consecutive 7 days [30]
  Group III (VAN + RC): VAN was injected I.P every 12 h at doses of 200 mg/kg/day, and RC extract was administrated by oral gavage at dose of 250 mg/kg/day (28) for consecutive 7 days.
  Group IV (VAN + RC): VAN was injected I.P every 12 h at doses of 200 mg/kg/day, and RC extract was administrated by oral gavage at dose of 500 mg/kg/day (28) for consecutive 7 days.
  Group V (RC group): RC extract was administrated by oral gavage for 7 consecutive days at dose of 500 mg/kg/day.

Twenty-four hours after the last injection, the blood samples were drawn by cardiac puncture to assess blood urea nitrogen (BUN), creatinine (Cr), nitric oxide (NO), malondialdehyde (MDA), and metabolites. All animals were sacrificed, and both the kidneys were removed and washed with ice-cold saline. The right kidney was kept in 10% formalin solution for histopathological examination, and the left was homogenized with Potter-Elvehjem in PBS (10%, w/v) (10 mmol/l, pH 7.4). The homogenate was centrifuged at 10000 ×g for 5 min at 4°C, the supernatant of renal tissues was stored at −20°C until use, and then, the levels of NO metabolites, MDA, protein carbonyl (PCO), and total thiols (tSH) content were determined.

### 2.4. Histological Evaluation

To evaluate histological changes, pieces of kidney tissue were fixed in 10% formalin. After four-step dehydration in an ascending ethanol series (70, 90, 96, and 100%), the right kidney was rinsed off with xylene and embedded in paraffin. Using a microtome, sections were taken and stained with hematoxylin-eosin according to the standard procedure [31]. The sections were examined under a light microscope by a pathologist unaware of the treatment group status. All the sections were examined at both low and high power magnifications. Histopathological renal tissues were evaluated for alterations in 4 components: tubules (dilatation, epithelial cell vacuolization and necrosis, casts), interstitium (inflammation, edema), glomeruli, and vessels. All these changes were rated as (−) showing no changes, mild (+) single cell necrosis and vacuolization, few foci of dilatation, casts, inflammatory infiltration, and edema, moderate (++) for all alterations at different foci throughout the tissue, and severe (+++) extensive and striking changes [32].

### 2.5. Biochemical Analysis

#### 2.5.1. Determination of BUN and Cr Levels

Using standard laboratory procedures, BUN and Cr levels were determined to analyze nephrotoxicity by commercial kits with an autoanalyzer (Olympus Instruments, Tokyo, Japan).

### 2.6. Oxidative Stress Markers

#### 2.6.1. Determination of MDA

Serum and tissue MDA levels, an indicator of lipid peroxidation, were performed based on the previous study [33]. The color was generated from the reaction between TBA and MDA at 535 nm. For serum and tissue, the MDA level was expressed as *μ*mol/L and nmol/g, respectively, using 1,1,3,3-tetramethoxypropane as standard.

#### 2.6.2. Determination of PCO Content

In tissue homogenates, total protein was determined by biuret reaction [34]. The color is produced by the reaction between DNPH and the carbonyls that strongly absorb light at 370 nm. All samples were diluted to protein concentration of 1 mg/mL with PBS. The level of the carbonyl group was estimated using a molar absorption coefficient of 2.2 × 10^4^ M^−1^cm^−1^ and demonstrated in tissue as *μ*mol/g [35].

#### 2.6.3. Determination of tSH

In tissue homogenate, using the spectrophotometric DTNB method, the content of total thiol was determined. With slight modifications of what described, DTNB measurement was performed [36]. Concisely, tissue homogenate (25 *μ*l) was mixed with Tris-EDTA buffer (150 *μ*l), 10 mM DTNB (10 *μ*l), and absolute methanol (790 *μ*l), in a microtube. However, test tubes were kept at room temperature for 15 min, and the absorbance determined at 412 nm. By using a molar absorption of 13,600 M^−1^cm^−l^, tSH groups were calculated.

#### 2.6.4. Determination of NO Metabolites

In tissue and serum, nitrite and nitrate levels were measured as an index of NO production in biological samples based on the Griess reaction method [37]. First, samples were deproteinized with acetonitrile (1 : 2, v/v), and then, 100 *μ*L of supernatant was added to a microplate well, followed by the addition of Griess reagent. After 30 min incubation at 37°C, the absorbance of samples was detected at 546 nm. The content of nitric oxide metabolites was determined from a linear standard curve defined by 0–100 *μ*mol/ml sodium nitrite. For tissue and serum, results were expressed as *μ*mol/g and *μ*mol/L, respectively.

### 2.7. GC-MS Analysis of the Extract

GC-MS analysis was carried out on a Hewlett-Packard 5973 linked with a mass detector HP6890 utilizing a DB-1 column (55 m × 0.25 mm, film thickness 0.25 *μ*m). The oven temperature was adopted from 40°C (1 min) to 250°C (30 min) at 3°C min^−1^. The transferor gas was helium with the flow rate of 1.0 ml min^−1^. The mass spectrometer (Hewlett-Packard 5973, USA) was activated in the EI mode at 70 eV.

### 2.8. Statistical Analyses

Results were analyzed by the one-way ANOVA test. Tukey's multiple comparison test was used to define statistical significance. The experiments were performed in duplicate, and the data were presented as mean ± standard error of the mean (SEM). The Kruskal–Wallis and Mann–Whitney *U*-tests were used to assess the pathology injury score between groups. For all tests, differences were considered significant at *P* < 0.05.

## 3. Results

### 3.1. BUN and Cr Levels in Serum

**Figure 1:**
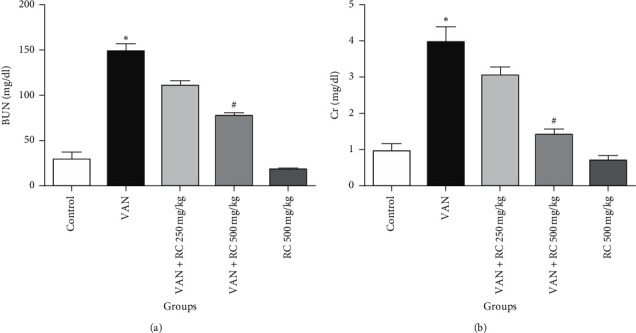
Effect of RC extract on kidney function markers in VAN-treated rat kidney. (a) BUN and (b) Cr. Each value represents the mean ± SEM. ^*∗*^Statistically significant compared to the control group (*P* < 0.05), ^#^statistically significant compared to the VAN group (*P* < 0.05). BUN, blood urea nitrogen; Cr, creatinine; VAN, vancomycin; RC, *Rosa canina*.

In serum, BUN and Cr levels were significantly increased in VAN-treated rats in comparison to the control group (*P* < 0.05). The hydroalcoholic extract of RC in the doses of 500 mg/kg significantly decreased BUN and Cr levels at *P* < 0.05 (Figure 1).

### 3.2. Oxidative Stress Markers in the Kidney

Results showed not only a significant reduction in tSH content but a remarkable increase in MDA, PCO, and NO metabolites in the VAN group compared to the control group (Table 1). A significant reduction in MDA, PCO, and NO metabolites was observed, while hydroalcoholic extract of RC at dose of 500 mg/kg was used in the VAN + RC group in comparison to the VAN-treated group (*P* ≤ 0.05). It should be noted that it had no significant effect on tSH content in the VAN + RC group compared to the VAN group.

### 3.3. Oxidative Stress Markers in Serum

Based on Table 2, MDA and NO levels were significantly increased in the VAN-treated group compared to control (*P* < 0.05). However, in VAN + RC 500 mg/kg, MDA and NO levels were significantly decreased compared to VAN-treated rats.

### 3.4. Histopathological Studies

**Figure 2:**
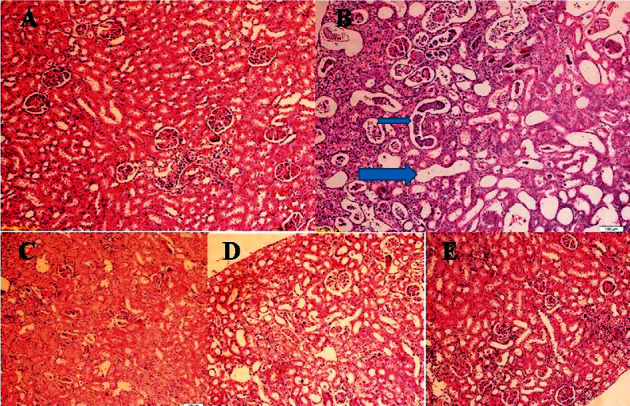
Photomicrograph of rat kidney stained with hematoxylin and eosin (10x). (A) Control. (B) Vancomycin (VAN) treated. (C) VAN plus 250 mg/kg hydroalcoholic extract of *Rosa canina*. (D) VAN plus 500 mg/kg hydroalcoholic extract of *Rosa canina*. (E) Only 500 mg/kg hydroalcoholic extract of *Rosa canina*. In the VAN group, diffuse tubular dilatation (large arrow) and casts (small arrow) are prominent at this picture. There are also areas of necrosis and inflammation. All of these changes are less prominent or absent in VAN + RC groups in C and D.

In the control group, histopathological examinations of the kidney did not show any abnormal morphology. However, in the kidneys of VAN-treated rats, tubular epithelial necrosis, vacuolization, tubular dilatation and casts, interstitial edema, dilatation of Bowman's space, and inflammatory cell infiltration were presented. Most of the changes had moderate to severe degree. As given in Table 3, oral treatment with RC extracts (250 and 500 mg/kg) decreased the interstitial nephritis, improved the renal tubule changes, and reduced the other morphological changes, in comparison to the VAN group. It should be noted that the structure of renal tissue in the VAN + RC (500 mg/kg) group was maintained near to normal (Figure 2).

### 3.5. GC-MS Components of the Hydroalcoholic Extract of RC

The chemical composition found in RC extract is given in Table 4. However, thirteen components have been identified with their percentage composition, characterized 100.0% of total RC. GC-MS analysis indicated that the main compositions were found to be lactose (21.96%), 3-t-butyloxaziridine (20.91%), 5-oxymethylfurfurole (16.75%), and 2-chlororesorcinol (7.29%).

## 4. Discussion

In the current study, the protective effect of *Rosa canina* on VAN-induced nephrotoxicity was investigated, since this compound is known as a highly effective scavenger for ROS [15, 22]. In clinical use, one of the most important complications of VAN is nephrotoxicity. Despite the introduction of new drugs into the pharmaceutical market, VAN still plays an important role in the treatment of MRSA and MRSE [13, 38]. The mechanism underlying VAN-induced nephrotoxicity remains unclear; however, oxidative stress is one of the l pathogenic mechanisms [11].

In various medical sciences, finding an appropriate approach to prevent or reduce the nephrotoxicity of VAN has always been a concern of researchers. In many countries, in addition to chemical drugs, the use of herbal medicines has been studied to reduce this complication due to less side effects, low toxicity, and reasonable prices. They have been used to protect against drug-induced toxicity and reinforce the endogenous antioxidants protections and repair the optimal balance by deactivating ROS [39]. Various studies have also been shown that RC fruit has a potent antioxidant effect in different tissues [16–18, 28].

A number of animal studies have suggested that VAN has oxidative effects on cells of the proximal renal tubules, and the use of antioxidants can prevent VAN-induced nephrotoxicity [11, 40]. According to the results of this study, administration of VAN induced a significant increase in the serum levels of BUN and Cr. These results are in agreement with other studies [41, 42] and indicate that the kidneys begin to fail even after few days of VAN administration. Also, histopathological findings showed that administration of VAN causes congestion, collapse of the glomerular structure, and dilation of various parts of the nephron, including the loop of Henle and proximal and distal convoluted tubules compared to the control group. Increased renal biomarkers associated with renal tubular damage support the role of oxidative stress in VAN-induced nephrotoxicity. These findings could be attributed to the accumulation of VAN in proximal tubules as reports have shown that VAN toxicity occurs in the proximal tubules and its vicinity [2, 3, 6]. Our results correspond well with those of the earlier studies that reported VAN-induced nephrotoxicity is related to oxidative stress [11, 32, 41, 43, 44].

*Rosa canina* at the dose of 500 mg/kg reduced the levels of Cr and BUN in plasma and reversed histological changes. Moreover, the results of the current study indicate potential inhibitory role of RC on VAN-induced nephrotoxicity.

The harmful effects of ROS can be explained by several mechanisms; they may produce cellular injury including peroxidation of membrane lipids, protein denaturation, and DNA damage [45–47]. The most sensitive compounds against oxidative stress are lipids. More importantly, results have confirmed the lipid peroxidation caused by free radicals playing a major role in VAN-induced oxidative stress [32, 43]. In the process of lipid peroxidation, MDA is generated by free radicals as a final product [3]. In the present study, MDA levels in serum and kidney, significantly increased in the VAN group compared to the control group. In agreement with other studies, the MDA levels increased duo to VAN nephrotoxicity [13, 40]. However, at dose of 500 mg/kg, RC extract decreased the elevated levels of MDA in serum and kidney which increased by VAN toxicity, indicating lipid peroxidation inhibited by RC. Of note, *Rosa canina* showed a renoprotective effect by decreasing lipid peroxidation, which is in line with a study which showed that RC prevented the lipid peroxide process in the kidney and liver [16, 48]. It is concluded that carotenoids in *Rosa canina* is a source of fat-soluble antioxidants and are in charge of inhibiting lipid peroxidation by not only quenching singlet molecular oxygen but also scavenging ROS [49, 50]. In a condition of ROS overproduction in the biological system, protein oxidation is an important outcome showing proteins are attacked by ROS [51]. Furthermore, to prove the severe oxidation of proteins, PCO is an indicator in biological samples [52]. In line with our results, in the mammalian system, there is growing evidence showing direct damage to proteins during oxidative stress can increase the PCO [51, 53]. Our results revealed that oral treatment with RC inhibited the PCO elevation due to VAN toxicity in renal. Moreover, PCO content slightly increased in the merely extract group as compared to the control group; may be, it is an unexpected effect of the extract. To measure tSH groups can be a good indicator to show the effects of oxidative stress on the proteins. Researchers have shown that hydroxyl and nitric oxide radicals can react with tSH groups and neutralize them. However, it seems tSH groups associated with small molecules like glutathione can effectively participate in these reactions [54]. In the tissue, high concentrations of glutathione protect vulnerable molecules against oxidizing agents. It should be noted that the level of tSH content indicates the antioxidant status of the body [55]. Therefore, the concentration of intracellular tSH groups are considered as a determinant key to estimate VAN-induced renal injury. It is clear that a reduction in renal tSH groups is one of the primary factors that provide a condition for lipid and protein peroxidation [13]. In our study, in rats receiving VAN, tSH groups were less compared to the control group, but not statistically significant and consistent with the results of other researchers. Our finding showed that in a VAN treated rats, the levels of nitric oxide metabolites were significantly increased in serum and kidney tissue compared to control. Moreover, an increase in renal nitric oxide levels by VAN was significantly suppressed by RC in rats treated by VAN + RC. Of note, the production of high nitric oxide in the VAN group may be related to the inflammatory response of the tissue [56, 57] and oxidative stress mediated by VAN [32, 41]. Therefore, in regards to the important role of this mediator, RC could inhibit VAN-induced nephrotoxicity via amelioration of oxidative stress and inflammation. One of the limitations of the current study was GC to determine the important components of the extract, and it was better we use LC mass for this aim.

## 5. Conclusion

Many evidences are supporting the role of oxidative stress as one of the important underlying mechanisms of VAN-induced nephrotoxicity. The oral administration of RC reduced the VAN-induced nephrotoxicity in rats by reducing lipid and protein peroxidation as well as the production of nitric oxide. These data suggested the potential of RC as a supplemental remedy for reducing renal toxicity-induced VAN. However, more investigations are needed to confirm these findings in clinical trial.

## Figures and Tables

**Table 1:** Effect of RC extract on oxidative stress markers in VAN-treated rat kidney.

Groups	tSH (*μ*mol/g tissue)	PCO (*μ*mol/g tissue)	NO metabolites (*μ*mol/g tissue)	MDA (*μ*mol/g tissue)
Control	18.65 ± 1.52	13.55 ± 1.01	28.83 ± 5.44	2.30 ± 0.34
VAN	12 ± 2.01^*∗*^	31.77 ± 2.13^*∗*^	53.61 ± 3.12^*∗*^	4.42 ± 0.16^*∗*^
VAN + RC 250 mg/kg	16.63 ± 2.01	30.37 ± 1.64	39.30 ± 1.93	3.22 ± 0.13^#^
VAN + RC 500 mg/kg	15.6 ± 1.21	22.97 ± 2.21^#^	33.69 ± 2.01^#^	2.31 ± 0.04^#^
RC 500 mg/kg	19.29 ± 2.19	20.47 ± 1.02	30.08 ± 1.78	2.50 ± 0.13

tSH, total thiol content; PCO, protein carbonyl; NO, nitric oxide metabolites; MDA, malondialdehyde; VAN, vancomycin; RC, *Rosa canina*. Each value represents the mean ± SEM. ^*∗*^Statistically significant compared to the control group (*P* < 0.05); ^#^statistically significant compared to the VAN group (*P* < 0.05).

**Table 2:** Effect of RC extract on oxidative stress markers in VAN-treated rat serum.

Groups	MDA (nmol/L)	NO metabolite (*μ*mol/L)
Control	2.57 ± 0.22	12.74 ± 0.29
VAN	4.03 ± 0.06^*∗*^	28.95 ± 1.58^*∗*^
VAN + RC 250 mg/kg	2.68 ± 0.12^#^	23.80 ± 0.99
VAN + RC 500 mg/kg	2.62 ± 0.09^#^	17.20 ± 0.48^#^
RC 500 mg/kg	2.74 ± 0.20	14.27 ± 0.22

NO, nitric oxide metabolites; MDA, malondialdehyde; VAN, vancomycin; RC, *Rosa canina*. Each value represents the mean ± SEM. ^*∗*^Statistically significant compared to the control group (*P* < 0.05); ^#^statistically significant compared to the VAN group (*P* < 0.05).

**Table 3:** Histopathological alteration scoring of renal tissue in experimental groups.

Histological parameters	Control	VAN	VAN + RC 250 mg/kg	VAN + RC 500 mg/kg	RC 500 mg/kg
Tubular epithelial necrosis	_	++	+	_	_
Vacuolization	_	+++	_	_	_
Tubular dilatation	_	+++	++	+	_
Tubular casts	_	++	+	+	_
Interstitial inflammation	_	+++	+	_	-
Interstitial edema	_	++	+	_	_
Dilatation of Bowman's space	_	+	_	_	_
Total histological score	0 ± 0	2.28 ± 0.28^*∗*^	0.85 ± 0.26^#^	0.28 ± 0.18^#^	0 ± 0^#^

VAN, vancomycin; RC, *Rosa canina*. Each value represents the group score mean. ^*∗*^Statistically significant compared to the control group (*P* < 0.05); ^#^statistically significant compared to the VAN group (*P* < 0.05).

**Table 4:** GC-MS analysis of the hydroalcoholic extract of RC.

NO	Compound	Molecular formula	RT (min)	Percentage (%)
1	Lactose	C₁₂H₂₂O₁₁	20.834	21.96
2	3-t-Butyloxaziridine	C_5_H_11_NO	17.944	20.91
3	5-Oxymethylfurfurole	C_6_H_6_O_3_	12.823	16.75
4	2-Chlororesorcinol	C_6_H_5_ClO_2_	10.587	7.29
5	9,17-Octadecadienal, (Z)-	C_18_H_32_O	29.172	6.24
6	1,14-Dibromotetradecane	C_14_H_28_Br_2_	43.358	5.40
7	Dihydro-3-methylene-5-methyl-2-furanone	C_5_H_8_O_2_	5.284	4.55
8	Benzyl (dideuterated) methyl ether	C_8_H_10_O	36.509	4.48
9	Palmitic acid	C_16_H_36_O_2_	26.511	3.17
10	2,6,10,14,18-Pentamethyl-2,6,10,14,18-eicosapentaene	C_25_H_42_	38.045	3.02
11	4H-Pyran-4-one, 2,3-dihydro-3,5-dihydroxy-6-methyl-	C_6_H_8_O_4_	6.306	2.55
12	14-Beta-H-pregna	C_21_H_36_	34.875	2.27
13	Di-(2-ethylhexyl) phthalate	C_24_H_38_O_4_	34.397	1.41
Total				100.0

## Data Availability

The data used to support the findings of this study are included within the article.
